# A radiomics nomogram for predicting postoperative recurrence in esophageal squamous cell carcinoma

**DOI:** 10.3389/fonc.2023.1162238

**Published:** 2023-10-12

**Authors:** Yahan Tong, Junyi Chen, Jingjing Sun, Taobo Luo, Shaofeng Duan, Kai Li, Kefeng Zhou, Jian Zeng, Fangxiao Lu

**Affiliations:** ^1^ Department of Radiology, Zhejiang Cancer Hospital, Hangzhou, China; ^2^ Medical School of Chinese People’s Liberation Army (PLA), Beijing, China; ^3^ Department of Thoracic Surgery, Zhejiang Cancer Hospital, Hangzhou, China; ^4^ GE Healthcare, Precision Health Institution, Shanghai, China; ^5^ Department of Radiology, The First Affiliated Hospital of Zhejiang Chinese Medical University, Hangzhou, China

**Keywords:** esophageal squamous cell carcinoma/esophageal cancer, radiomics, tomography, X-Ray Computed, nomogram, recurrence

## Abstract

**Purpose:**

To establish and validate a radiomics nomogram for predicting recurrence of esophageal squamous cell carcinoma (ESCC) after esophagectomy with curative intent.

**Materials and methods:**

The medical records of 155 patients who underwent surgical treatment for pathologically confirmed ESCC were collected. Patients were randomly divided into a training group (n=109) and a validation group (n=46) in a 7:3 ratio. ​Tumor regions are accurately segmented in computed tomography images of enrolled patients. Radiomic features were then extracted from the segmented tumors. We selected the features by Max-relevance and min-redundancy (mRMR) and least absolute shrinkage and selection operator (LASSO) methods. A radiomics signature was then built by logistic regression analysis. To improve predictive performance, a radiomics nomogram that incorporated the radiomics signature and independent clinical predictors was built. Model performance was evaluated by receiver operating characteristic (ROC) curve, calibration curve, and decision curve analyses (DCA).

**Results:**

We selected the five most relevant radiomics features to construct the radiomics signature. The radiomics model had general discrimination ability with an area under the ROC curve (AUC) of 0.79 in the training set that was verified by an AUC of 0.76 in the validation set. The radiomics nomogram consisted of the radiomics signature, and N stage showed excellent predictive performance in the training and validation sets with AUCs of 0.85 and 0.83, respectively. Furthermore, calibration curves and the DCA analysis demonstrated good fit and clinical utility of the radiomics nomogram.

**Conclusion:**

We successfully established and validated a prediction model that combined radiomics features and N stage, which can be used to predict four-year recurrence risk in patients with ESCC who undergo surgery.

## Introduction

Esophageal cancer is the seventh-most prevalent cancer and has the sixth-highest overall mortality rate among all malignancies (1). Furthermore, esophageal cancer is one of the deadliest and most invasive of all gastrointestinal cancers (2), and approximately half of all esophageal cancer patients experience postoperative recurrence (3). Surgery remains the most effective treatment, especially for early-stage patients. However, recurrence is the primary cause of treatment failure (4). Moreover, recurrence usually occurs within 2 years of the end of treatment (5, 6). Once relapse occurs, patients usually have an unfavorable prognosis, with a reported survival duration of 3–10 months (7). Accurately predicting postoperative recurrence and offering preventive treatment measures is therefore an urgent issue to be addressed in clinical practice. Clinical stage is an important factor that affects prognosis. However, because of the heterogeneity of tumors, patients with the stage of disease have significant variation in prognosis (8). Therefore, early and accurate identification of these patients is beneficial for designing individualized treatments and improving prognosis.

For patients with esophageal squamous cell carcinoma (ESCC), the pre-treatment clinical Tumor-Node-Metastasis (TNM) staging continues to be used widely for predicting prognosis (9). However, the current method has several limitations: the criteria for clinical TNM stage are the same as those for pathological stage, which is based on imaging assessments of lesion size and invasion of peripheral organs; thus, high-dimensional medical imaging data is ignored.

Computed tomography (CT) is a widely used imaging modality that provides a large number of quantitative features and fine anatomical structures that are valuable for confirming the presence of esophageal cancer. CT is the most common non-invasive imaging tool for lesion assessment. In addition, the evaluation of tumor heterogeneity is of great significance for evaluating the degree of malignancy and predicting the prognosis of patients. CT-based imaging informatics has developed rapidly over the last several years and provide valuable information for diagnoses and predictions of prognosis. Driven by the trend of artificial intelligence, “radiomics” was proposed, which involves the rapid extraction of numerous quantitative features from tomographic images via high-throughput computation, and the digital medical images are subsequently converted into mineable multidimensional data (10). This method allows the exploration of pathophysiological information of various diseases using medical images, and the association between images and prognosis can be analyzed (11, 12). Previous studies have reported that radiomics has the potential to predict therapeutic response and prognosis in ESCC patients. Xie et al. suggested that radiomics is superior to volumetric measurements for disease assessment and that it can provide valuable predictions for individualized overall survival (13). Wu et al. combined radiomic features and clinical risk factors to construct a radiomic model and found that it could predict lymph node (LN) metastasis in ESCC patients before surgery (14). Lu et al. indicated that the dual-region radiomics signature is an independent prognostic marker that is better than the single-region signature for predicting ESCC patients’ overall survival (OS); moreover, combining the dual-region radiomics signature and clinicopathological factors could further improve OS prediction (15).

Recently, several clinicopathologic biomarkers have been confirmed as valuable for the prediction of therapeutic response and prognosis in patients with ESCC (16–18). Therefore, we aimed to develop and validate a radiomics nomogram based on a radiomics signature and clinical independent predictors for the prediction of recurrence in ESCC patients who have undergone surgical treatment with curative intent.

## Materials and methods

### Patients

The study protocol was approved by the Ethics Committee of the hospital. During the study, ESCC patients who received radical treatment in our hospital from January 2015 to November 2016 were enrolled. Based on the relevant criteria, 155 patients met the requirements and were included in the study. All selected subjects underwent chest-enhanced CT examination before the operation.

The inclusion criteria were as follows (1): postoperatively and pathologically confirmed ESCC; (2) contrast-enhanced CT of the chest performed within one month before surgery; and (3) no distant metastases prior to surgery. The exclusion criteria were as follows: (1) incomplete clinical information; (2) receipt of tumor-related treatment (e.g., chemotherapy or radiotherapy) prior to undergoing CT; (3) poor CT image quality or unrecognizable lesion; and (4) other concurrent malignancies. Patient clinical data, which included sex, age, and TN stage, were obtained from medical records following surgery. Tumor location was determined based on the 8th edition of the AJCC Cancer Staging Manual (19). Follow-up and survival data were collected based on telephonic inquiries. Recurrence was confirmed by histopathological biopsy or clinical follow-up, and included locoregional, distant, or a combination of both. The time of recurrence started from the day of the operation to the discovery of recurrence. Each patient was followed-up for at least 4 years or until the time recurrence occurred. Patients with esophageal cancer were divided into the recurrence group (recurrence occurred in 4 years) and non-recurrence group (recurrence did not occur in 4 years).

### CT image acquisition

In the course of imaging examination, all subjects underwent chest-enhanced CT scanning through multi-detector CT system: Bright Speed, Optima CT 680 Series (GE Medical Systems), Siemens Somatom definition AS 64, and Perspective (Siemens Medical Systems). The scanning parameters set during inspection are as follows: detector configuration 128×0.6 mm; tube voltage, 120–130 kV; tube current, 150–300 mAs; thickness, 5 mm; and pitch, 0.6. According to the obtained image, tumor segmentation and feature extraction were performed.

### Tumor segmentation

**Figure 1:**
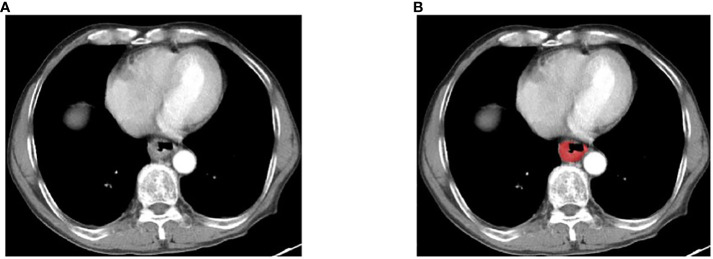
An example of manual segmentation in ESCC. **(A)** Localized thick wall of esophageal cancer with enhancement is observed on the arterial phase computed tomography (CT) image; **(B)** Manual segmentation on the same axial slice is depicted with red label.

Pre operative enhanced CT images were collected and saved based on a unified format. Two doctors with extensive experience in imaging examination of digestive system diseases observed the images, and the CT images of each layer were compared and analyzed in detail. Tumor regions of interest (ROIs) were delineated using ITK-SNAP (http://www.itksnap.org); an example is shown in **Figure 1**. For the tumor ROIs, radiologists reviewed all CT image slices of each patient and segmented the three-dimensional-labeling ROIs covering the whole entire tumor. Observer 1 delineated the lesions of ESCC. The observer 2 re-checked the tumor segmentation area. If the assessment of two radiologists was inconsistent, a thorough negotiation was conducted until consensus was reached.

### Radiomics feature extraction and selection

Radiomics feature extraction was based on Pyradiomics (https://pyradiomics.readthedocs.io/en/latest/), which is an efficient, open-source platform, through which users can process medical images to extract radiomics features. In this paper, the maximum correlation minimum redundancy (mRMR) and LASSO algorithms were used to select the features, and the rad-score of every ESCC patient was calculated using their coefficients.

### Construction of the predictive model

For the construction of the radio-clinical model, we first used univariate analysis to analyze the clinical predictors and rad-score, and the strongly associated features were then processed in the multivariate logistic regression analysis. The multivariable logistic regression analysis was used to develop a prediction model by combining the rad-score and clinical predictors (P<0.05). In the training cohorts, for the sake of convenience, the model was converted to the radio-clinical nomogram. The performance of the radio-clinical model was tested by the validation set.

### Performance of the radio-clinical nomogram

The ROC curve, calibration curve, and Decision curve analysis (DCA) were used to evaluate the prediction of this radio-clinical model. During validation, the performance of this model was evaluated by 10-fold cross validation, and the best model was obtained by comparing the results. The diagnostic value of the clinical model was proved using the validation set. In the evaluation process, the area under the ROC curve (AUC), sensitivity, and specificity-related indicators were calculated first. We also used the Delong test to compare the AUC values in different models. DCA mainly verifies the clinical practicability of the model through net income.

### Statistical analysis

The statistical analyses were carried out using the R software (http://www.Rproject.org). Quantitative data were described as means ± standard deviations, and qualitative data were described as frequencies (percentages). The independent predictive factors in the variables are determined based on multiple regression analysis. Significant difference was based on P<0.05. The “glmnet” package was used in LASSO regression analysis, and the “rms” package was used in multivariate regression analysis. The “pROC” package was used to process the collected data and establish the ROC diagram. “Rmda” package was used for DCA analysis.

## Results

### Clinical characteristics

Based on the statistical analysis, 93 of the 155 patients with ESCC were in the recurrence group and 62 were in the non-recurrence group. Of the 155 patients included in this study, 93 patients developed disease progression within 4 years. In both the training and validation sets, higher rad-scores were found in the recurrence group than in the non-recurrence group. Additional details are provided in **Table 1**.

**Table 1:** Clinic-radiological characteristics of patients in training and validation cohorts.

Characteristic	Training cohort		P-value	Validation cohort		P-value
	recurrence (n=63)	non-recurrence (n=46)		recurrence (n=30)	non-recurrence (n=16)	
Age(Y)			0.726			0.021
mean (sd)	62.3 (6.9)	62.8 (8.4)		60.7 (7.8)	65.4 (2.9)	
Sex			0.993			0.987
Male	56 (88.9)	40 (87.0)		24(80.0)	12 (75.0)	
Female	7 (11.1)	6 (13.0)		6 (20.0)	4 (25.0)	
T stage			0.027			0.313
T1	6 (9.5)	13 (28.3)		3 (10.0)	2 (12.5)	
T2	13 (20.6)	8 (17.4)		6 (20.0)	7 (43.8)	
T3	36 (57.1)	24 (52.2)		16 (53.3)	6 (37.5)	
T4	8 (12.7)	1 (2.2)		5 (16.7)	1 (6.2)	
N stage			<0.001			0.109
N0	14 (22.2)	34 (73.9)		15 (50.0)	13 (81.2)	
N1	30 (47.6)	9 (19.6)		7 (23.3)	3 (18.8)	
N2	16 (25.4)	2 (4.3)		6 (20.0)	0 (0.0)	
N3	3 (4.8)	1 (2.2)		2 (6.7)	0 (0.0)	
Location			0.286			0.832
Low	25 (39.7)	12 (26.1)		10 (33.3)	4 (25.0)	
Middle	35 (55.6)	30 (65.2)		18 (60.0)	11 (68.8)	
Upper	3 (4.8)	4 (8.7)		2 (6.7)	1 (6.2)	
Rad-sore			< 0.001			0.004
median [iqr]	0.8 [0.3, 1.1]	-0.1 [-0.8, 0.3]		0.8 [0.3, 1.6]	-0.1 [-0.4, 0.5]	

### Radiomics feature selection and radiomics signature construction

**Figure 2:**
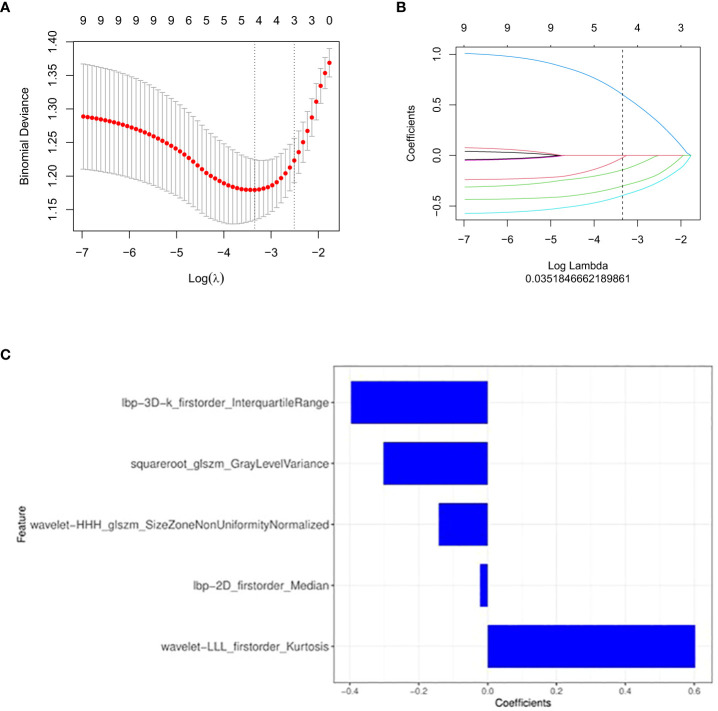
Feature selection with the least absolute shrinkage and selection operator (LASSO) binary logistic regression model. **(A, B)** The LASSO includes choosing the regular parameter λ, determining the number of the feature. **(C)** The selected radiomics features (with nonzero coefficients) and their coefficients.

**Figure 3:**
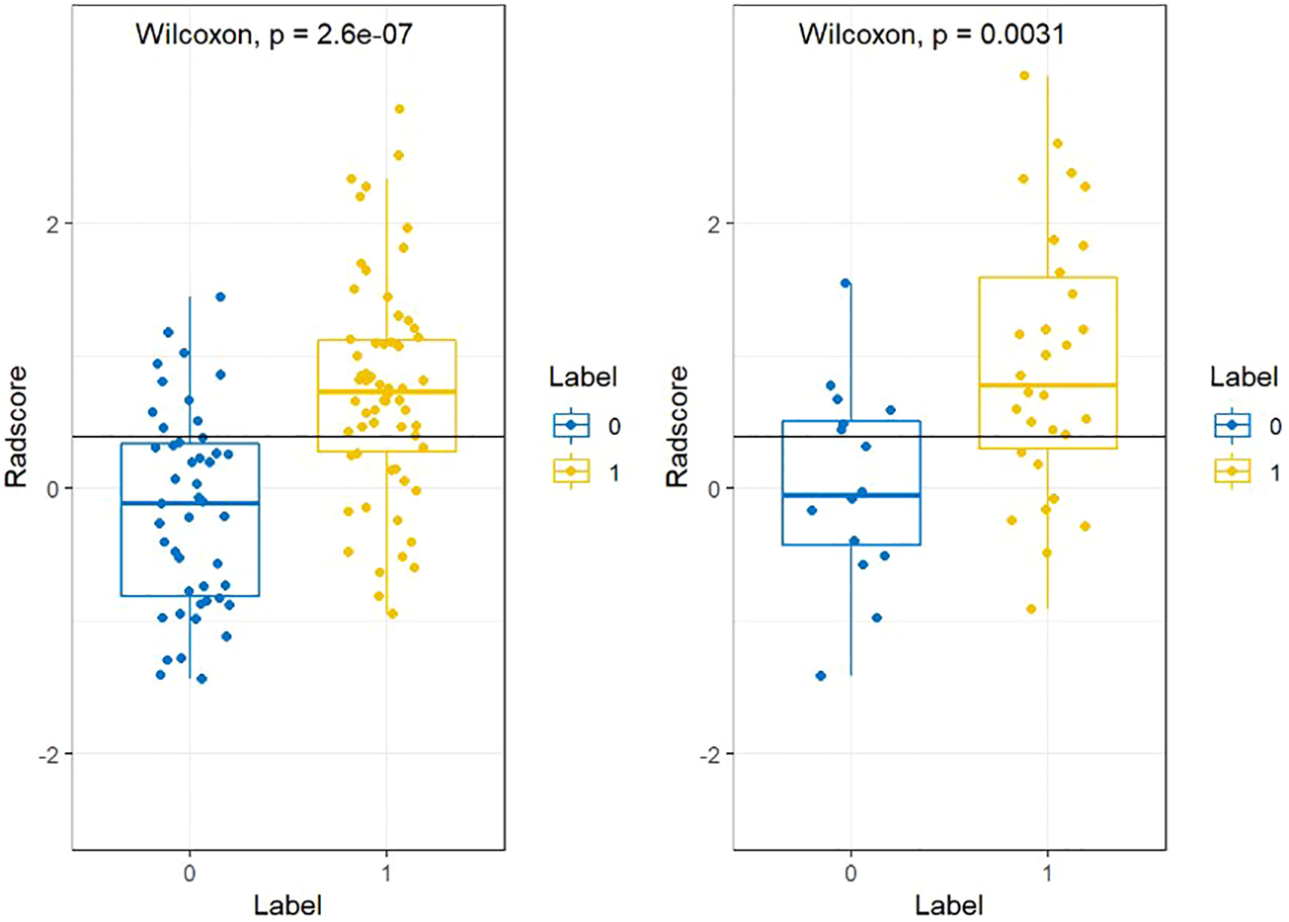
The radscores from class 0 and class 1 on training group and testing group respectively. “0” for no recurrence, “1” for recurrence.


After preprocessing the CT images of each patient, 1781 radiomics features were extracted. At the beginning of processing, redundant and meaningless features were deleted through mRMR, and 30 features were obtained after processing. Then, an optimized feature subset was selected based on LASSO, and the model was established after appropriate processing. When selecting the best radiomics features, LASSO method with 10-fold cross validation is applied to process the results, as shown in **Figure 2**. Finally, the model contained five radiomics features, which were weighted by coefficients to obtain the rad-score, as shown in **Figure 3**. The corresponding calculation expression is as follows:

$$\begin{array}{l} Radscore=-0.022*lbp\_2D\_firstorder\_Median-0.142\\ {}*wavelet\_HHH\_glszm\_SizeZoneNonUniformityNormalized\\ +0.603*wavelet\_LLL\_firstorder\_Kurtosis-0.397\\ {}*lbp\_3D\_k\_firstorder\_InterquartileRange-0.302\\ {}*squareroot\_glszm\_GrayLevelVariance+0.387 \end{array}$$

### Development of a simplified radiomics nomogram

**Figure 4:**
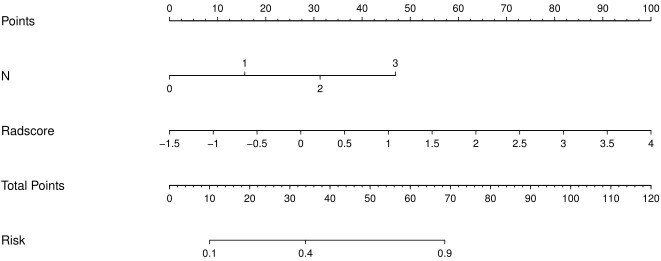
The CT-based radiomics nomogram. The radiomics nomogram was built in the training cohort, with the radiomics signature and N stage.


The results of univariate analysis show that T stage, N stage and radiomics signature served as the risk factors of postoperative recurrence in ESCC patients. After multivariate logistic regression analysis, N stage and radiomics signature were identified as independent predictors of postoperative recurrence in ESCC patients (**Table 2**). Multivariable analysis was performed to develop a prediction model by combining the rad-score and N stage. The radiomics nomogram is illustrated in **Figure 4**. The formula for the nomoscore is as follows:

**Table 2:** Predictors for recurrence status in ESCC.

Variable	Odds Ratio (95%CI)	p-value
N	3.23 (1.66-6.30)	< 0.001
Radscore	3.91 (1.94-7.88)	< 0.001

Nomoscore = −0.902+N*1.173+Radscore*1.363

### Performance of the radiomics nomogram

**Figure 5:**
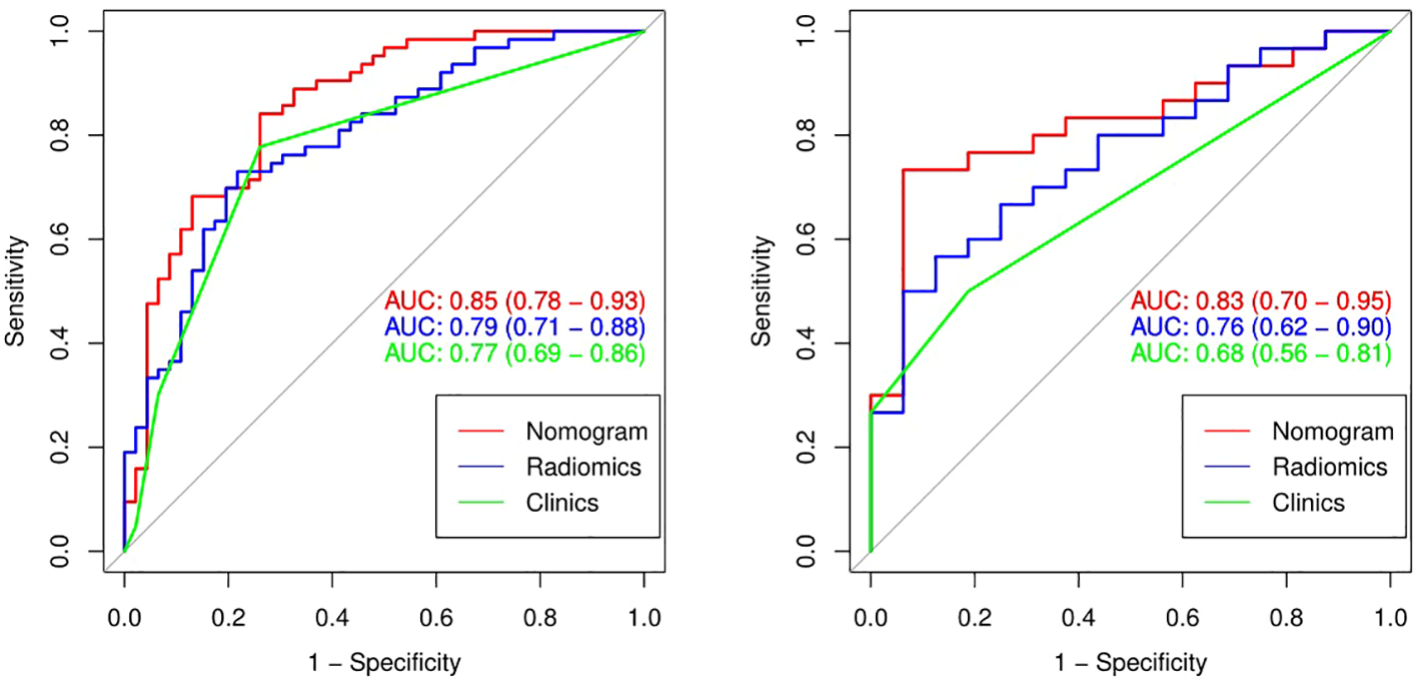
The ROC curves (AUC) of the three models in the training set **(A)** and the validation set **(B)**.

**Figure 6:**
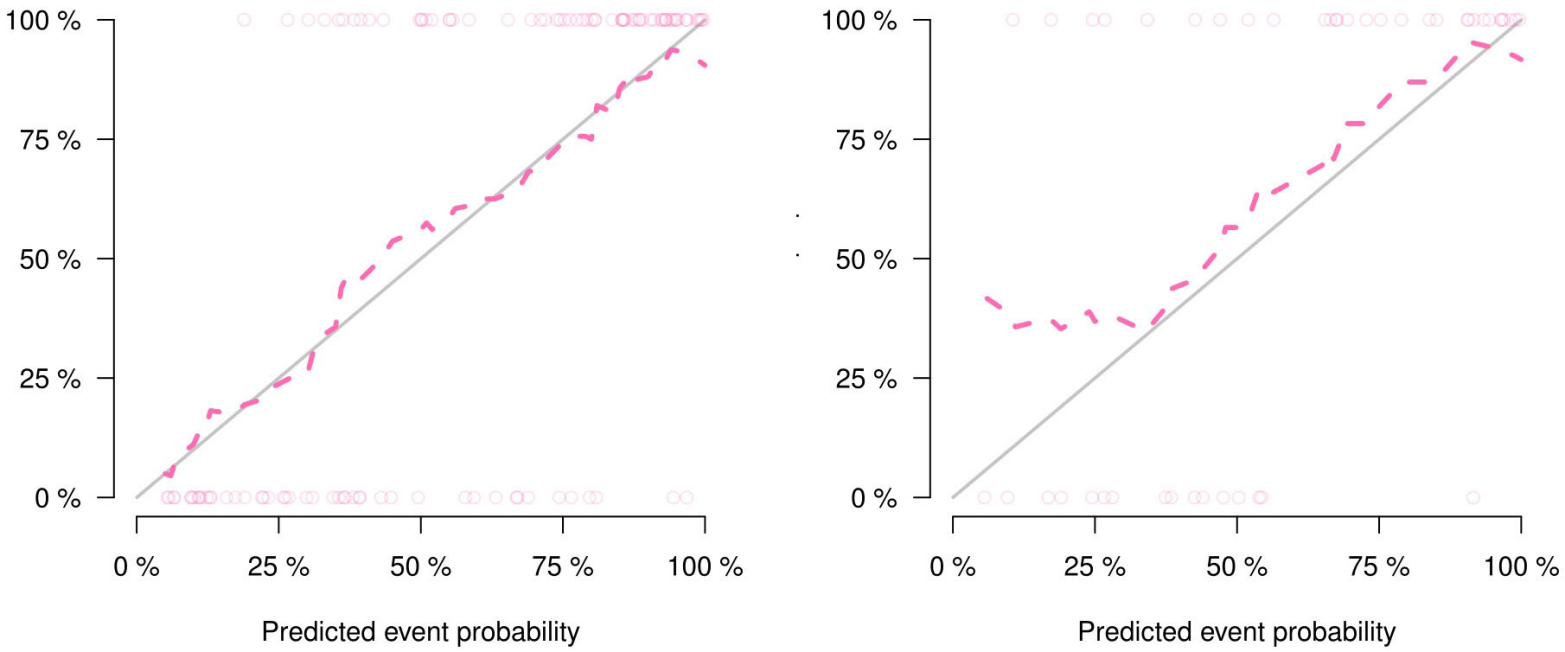
Calibration curves of the nomogram in the training set **(A)** and the validation set **(B)**.

**Figure 7:**
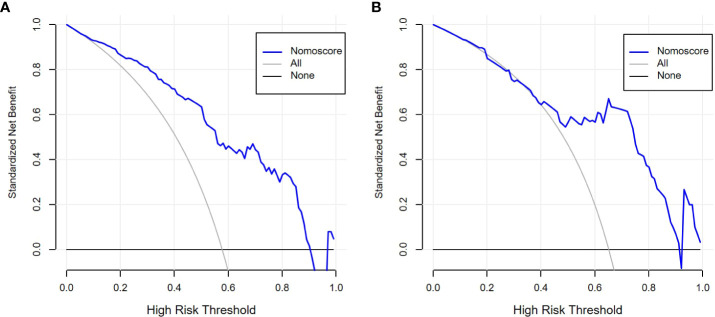
Decision curve analysis (DCA) of radiomics nomogram in the training set **(A)** and validation set **(B)**.

The performance comparison results of the radiomics nomogram are shown in **Table 3**. According to the results in **Figure 5**, the prediction ability based on the radiomics features model is limited. The AUC values of this model were 0.79 and 0.76 in the training set and validation set, respectively. If the prediction is only based on the clinical characteristics, the corresponding AUC values are 0.77 and 0.68, respectively. The model combining radiomics features and clinical factors has stronger performance than other relevant models and can effectively predict the recurrence risk in the application process. According to **Figure 5**, the AUC values of this model for the training set and validation set are 0.85 and 0.83, respectively. The calibration curve of the radiation nomogram also showed good prediction performance (**Figure 6**). We used DeLong’s test to compare whether the ROC curves are different between nomogram and clinical model. The DeLong’s test showed that the statistical difference between the nomogram and clinical model was significant (P = 0.006 for the training cohort and P =0.019 for the validation cohort). The radiomics nomogram DCA showed a higher overall net benefit than the clinical factors model, demonstrating high clinical utility in predicting postoperative recurrence (**Figure 7**).

**Table 3:** Predictive performance of radiomics nomogram.

Radiomics nomogram	AUC(95%CI)	Accuracy	Sensitivity	Specificity	PPV	NPV
Training cohort	0.85(0.78-0.93)	0.798	0.841	0.739	0.815	0.773
Validation cohort	0.83(0.70-0.95)	0.761	0.852	0.632	0.767	0.750

## Discussion

Surgery is the most effective strategy for treating early esophageal cancer (20). However, the rate of local recurrence with surgical treatment alone remains high (7). Accurate identification of patients prone to relapse is important for individualized treatment options. CT is a widely accepted clinical imaging modality and occupies an important position in the diagnosis, staging, and response evaluation of esophageal cancer (21). In this study, we used enhanced CT images before surgical treatment to establish a radio-clinical model to predict recurrence in patients with esophageal cancer. CT images are useful in predicting the prognosis of esophageal cancer after surgical treatment, yet poor in revealing potential tumor heterogeneity. Radiomics based on medical images is an emerging method for predicting cancer treatment response and long-term survival. To date, numerous studies have demonstrated the significant value of radiomics in clinical practice. Gillies et al. reported the great potential of radiomics to distinguish between benign and malignant diseases and predict the prognosis of tumor patients (11). Moreover, Ganeshan et al. confirmed that CT texture analysis can evaluate the heterogeneity of esophageal cancers (22).

The radiomics model in our study was established using five radiomics features and achieved a moderate result in predicting recurrence of esophageal cancer after surgery in both the training and validation sets. Two features were wavelet-based features, similar to that reported by few other studies (23–25). Wavelet transform is a new analysis technique developed from the boundedness of the short-time Fourier transform; however, it made up for its deficiencies (e.g., it can provide a change with the frequency of the “time-frequency” window) and is the best solution for analyzing and processing signal time-frequency (26). The “rad-score” integrated multiple radiomics features into a biomarker using multivariate logistic regression models. Our study suggested that the rad-score is an independent predictor of recurrence in ESCC patients after surgical treatment. In contrast to the “N stage,” radiomics features were the dominant factor in our radiomics nomogram (27).

Our radiomics model can predict postoperative recurrence to a certain extent. However, clinical characteristics, such as tumor location, T stage, and N stage, are also important influencing factors of esophageal cancer postoperative recurrence (28). These clinical factors are easily identified during the course of treatment and do not further burden patients. Over the past few years, a growing number of studies have shown that combining radiomics markers with clinical factors improves the accuracy of disease prediction (23, 29). Therefore, we hypothesized that our radiological model could improve predictive performance when combined with clinical factors, which we verified experimentally. Our radiomics signature contained five relevant radiomics features and offered moderate predictive efficacy. The AUC values of the radiomics model in the training and validation sets were 0.79 and 0.76, respectively. When we integrated the independent clinical risk factors with the radiomics signature, the predictive power of the model improved. The AUC values of the radiomics nomogram in the training and validation sets were 0.85 and 0.83, respectively, which showed that the performance of our radiomics nomogram was superior to that of both the radiomics feature model and the clinical factor model.

In terms of clinical factors, we included the postoperative T stage and N stage to avoid the deviation in prediction results caused by inaccurate judgments of TN stage owing to the subjective differences between radiologists. All patients included in this study had esophageal cancer who had undergone surgical resection; hence, none of the patients developed distant metastasis. Therefore, the clinical factors included in this study did not consider the difference in M stage. Furthermore, we found that there was no statistical difference in T stage between the two groups of patients, regardless of relapse. The reason for this might be because the primary tumor lesion was completely removed after radical resection of esophageal cancer, so the degree of invasion of the primary tumor would not be an independent risk factor for recurrence. Lymph node metastasis is used extensively to stratify ESCC patients according to the risk of recurrence. This is important for identifying patients who are likely to benefit from neoadjuvant chemoradiation (30). Previous studies have verified that lymph node metastasis is an independent risk factor for recurrence. In our study, univariate analysis showed that N stage was an independent predictor of recurrence risk; however, there were no significant differences between the recurrence and non-recurrence groups with respect to sex, age, T stage, or tumor location. Therefore, we included N stage in our prediction model.

In the present study, we constructed and validated a radiomics nomogram for the prediction of postoperative 4-year recurrence risk in patients with ESCC who have undergone surgery. The user-friendly nomogram comprising a radiomics signature and N stage demonstrated excellent performance in both cohorts and accurately stratified patients according to postoperative recurrence risk. The nomogram was built using a well-calibrated and well-validated prediction model. Our findings supported our hypothesis in that patients can be successfully stratified using a radiomics nomogram that integrates radio-clinical features by showing good performance in both the training and validation cohorts.

This study has some limitations. First, because long-term prognostic follow-up information was not readily available, the sample size of our study was small. Moreover, it was a single-center study. Thus, it is essential to conduct further large-scale and multi-center studies. Second, because of the retrospective nature of the study, there may be some bias. In future research, a time-divided model should be established. Finally, several previous studies have combined genetic information with radiomics features to predict prognosis; however, we did not include genetic information in this study. Future studies should aim to incorporate genetic information into radio-clinical features.

## Conclusions

Our prediction model was established by combining radiomics features with N stage, and it shows great promise and clinical application value for predicting the 4-year recurrence of ESCC following surgery.

Declarations

## Data availability statement

The data analyzed in this study is subject to the following licenses/restrictions: The original data for this article are not publicly available because of patients information privacy. Requests to access these datasets should be directed to Fangxiao Lu (lfx80611@163.com).

## Ethics statement

This retrospective study was approved by the Medical Ethics Committee of Zhejiang Cancer Hospital (No. IRB-2022-54). The Medical Ethics Committee of Zhejiang Cancer Hospital waived the need for informed consent.

## Author contributions

YT completed the initial manuscript and designed the whole study; JC collected imaging data and participated in revising the manuscript; JS and KL collected imaging data and participated in tumor segmentation; TL collected patients and recorded the needed information; SD was responsible for data processing and interpretation; KZ participated in the statistics and provided result interpretation and revising the manuscript; JZ and FL revised the manuscript and guaranteed the entire study. All authors contributed to the article and approved the submitted version.
